# Supplementary material for: Response Surface Methodology-Based Optimization of the Chitinolytic Activity of Burkholderia contaminans Strain 614 Exerting Biological Control against Phytopathogenic Fungi
Source: Microorganisms. 2024 Aug 2;12(8):1580. doi: 10.3390/microorganisms12081580 (PMC11356717; doi:10.3390/microorganisms12081580)
Supplement: Supplementary file 1 [file microorganisms-12-01580-s001.zip › microorganisms-3032981-supplementary.pdf]

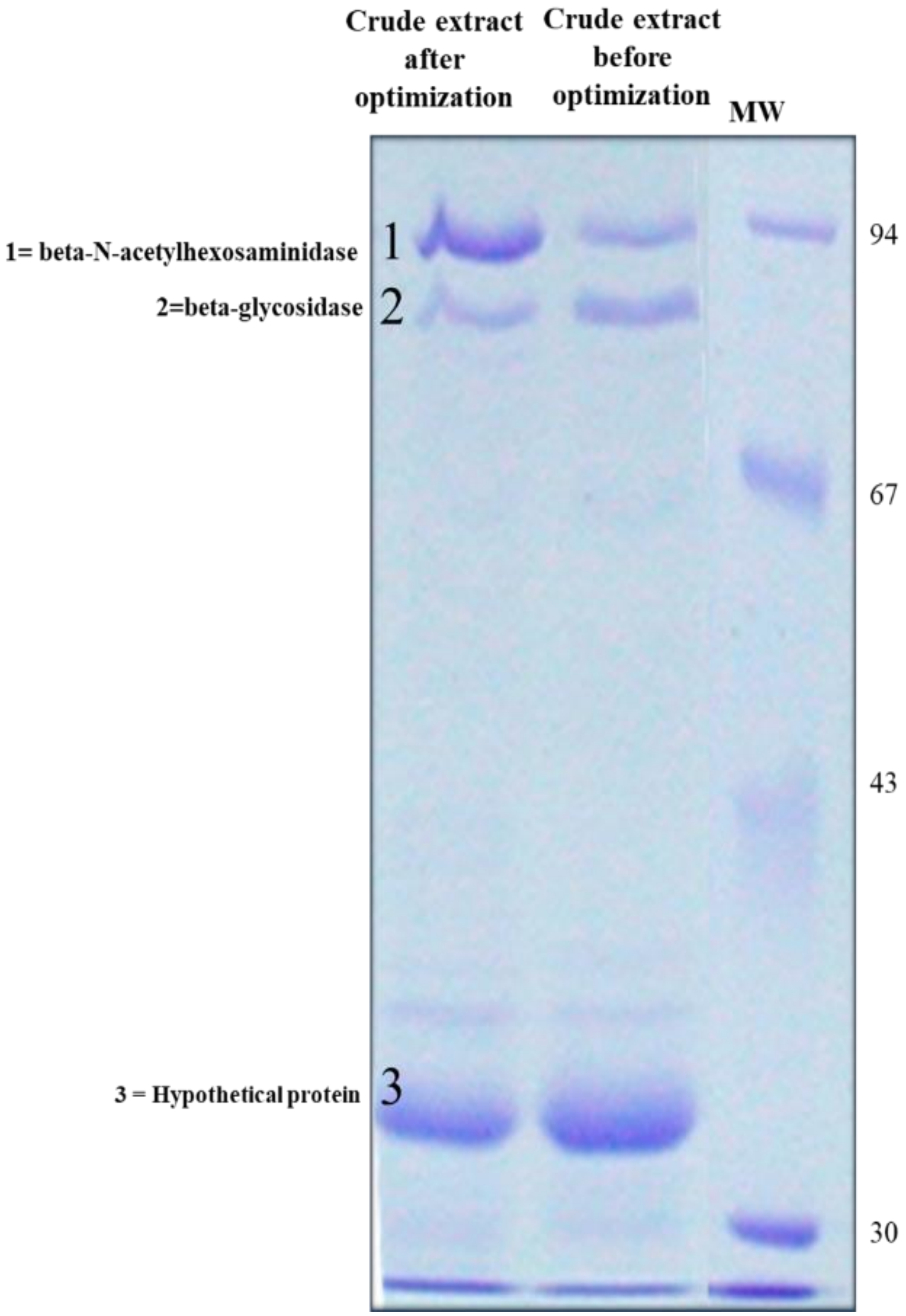

Figure S1. SDS-PAGE electrophoresis of the crude extract containing chitinase before and after optimization
